# Association Between Polymorphisms in DNA Repair Genes and Glioma Susceptibility: A Meta-Analysis of Four Single Nucleotide Polymorphisms (rs3212986, rs13181, rs25487, and rs861539)

**DOI:** 10.7759/cureus.76084

**Published:** 2024-12-20

**Authors:** George Fotakopoulos, Mohamed M Montasr, Vasiliki E Georgakopoulou, Charalabos Gatos, Nikolaos Foroglou

**Affiliations:** 1 Department of Neurosurgery, Aristotle University of Thessaloniki, AHEPA University Hospital, Thessaloniki, GRC; 2 Department of Neurosurgery, General University Hospital of Larissa, Larissa, GRC; 3 Department of Pathophysiology and Pulmonology, Laiko General Hospital, Athens, GRC

**Keywords:** brain tumors, gene associations, glioblastoma, glioma, snps

## Abstract

Cases with central nervous system tumors represent a small amount of all tumors, and the diagnosis of high-grade gliomas (HGGs) is mostly difficult as they frequently show intratumoral morphological heterogeneity. Genetic factors, such as single nucleotide polymorphisms (SNPs), have an important role in modifying glioma susceptibility. We conducted a comprehensive meta-analysis to investigate the ERCC1 (rs3212986), ERCC2 (rs13181), XRCC1 (rs25487), and XRCC3 (rs861539) genes to see if they are any risk factors for glioma susceptibility. We identified 30 eligible studies investigating the PubMed records (up to January 2024) via a mishmash of the subsequent terms: brain tumors, glioma, glioblastoma, gene associations, SNPs, XRCC1, XRCC3, ERCC1, and ERCC2. The total number of patients was 23678 (9731 in cases (poor outcome) and 13947 in controls (good outcome)). This comprehensive meta-analysis declared a significant association between ERCC2 rs13181, XRCC1 rs25487, and the risk of glioma.

## Introduction and background

Cases with central nervous system (CNS) tumors constitute around 2% of the entire tumors, with an expected 4.2 to 5.4 per 100,000 entities for each year [1]. Even though the number of CNS cancers is smaller than other malignancies, they constitute one of the main human cancers, as they impact the organization and incorporation of the entire organic functions. In addition, CNS cancer complications such as seizures can be the first presenting symptom before a diagnosis is confirmed in approximately 30%-60% of primary brain tumor patients [2]. Furthermore, as every location of the CNS has a critical role, the management performed in other neoplasms (extending surgical excision of the organ or cancer to a safe border of healthy tissue) wouldn’t be practical to treat brain neoplasms [1].

The 2016 World Health Organization (WHO) Classification of Tumors of the Central Nervous System [3] divides brain neoplasms, which consist of more than 40 diverse entities, into six main groups. In the fifth edition of the WHO 2021 Classification, 14 newly recognized types have been added to the classification of gliomas, glioma, and neuronal tumors [4].

Diagnosing high-grade gliomas (HGGs) is mostly difficult as they frequently show intratumoral morphological heterogeneity. As gliomas encompass a diverse range of CNS cancers, including astrocytomas, oligodendrogliomas, and ependymomas - historical designations that reflect the putative tissue of origin, glioblastoma, the most aggressive variant, constitutes a part of HGGs [4]. Lately, it has been shown that the recognition of biomarkers prognostic of the outcome of cases would be a key door to progress in the identification of HGGs [5]. Currently, the etiology of gliomas has not been completely understood. Previous research has shown that an amount of single nucleotide polymorphisms (SNPs) in the DNA repair gene may alter glioma danger [6-14].

Genetic elements, like SNPs, comprise an essential function in altering glioma risk, such as excision repair cross-complementation group 1 (ERCC1), SNPs in X-ray repair cross-complementing protein 1, vascular endothelial growth factor (VEGF) genes, and interleukin-8 [15-17]. Also, the rs13181 polymorphism of excision repair cross-complementation group 2 (ERCC2) is a T-to-G replacement at the 751 locus, which probably modifies the enzymatic action of the determined protein. ERCC2 rs13181 is connected with different malignancies, like esophageal and gastric cancer, hepatocellular carcinoma, non-small cell lung and skin cancer, bladder cancer, and prostate cancer [18-22].

Polymorphisms in DNA repair genes leading to variations in DNA repair efficiency may be correlated with the development of several kinds of cancers [23,24].

The authors have proposed that genes involved in the cell cycle and DNA restoration have a crucial involvement in glioma development, like glutathione S-transferases (GSTs), excision repair cross-complementing rodent repair deficiency complementation group 1 (ERCC1), X-ray repair cross-complementing groups 3 (XRCC3), and X-ray repair cross-complementing groups 1 (XRCC1) [25-27].

However, the clinical value of most glioma-associated molecular aberrations in terms of their significance as diagnostic, prognostic, or predictive molecular markers has remained unclear [28-30].

We conducted a comprehensive meta-analysis with the aim of investigating the ERCC1 (rs3212986), ERCC2 (rs13181), XRCC1 (rs25487), and XRCC3 (rs861539) genes to see if there are any risk factors for glioma susceptibility.

## Review

Materials and methods

Study Identification and Selection

**Figure 1:**
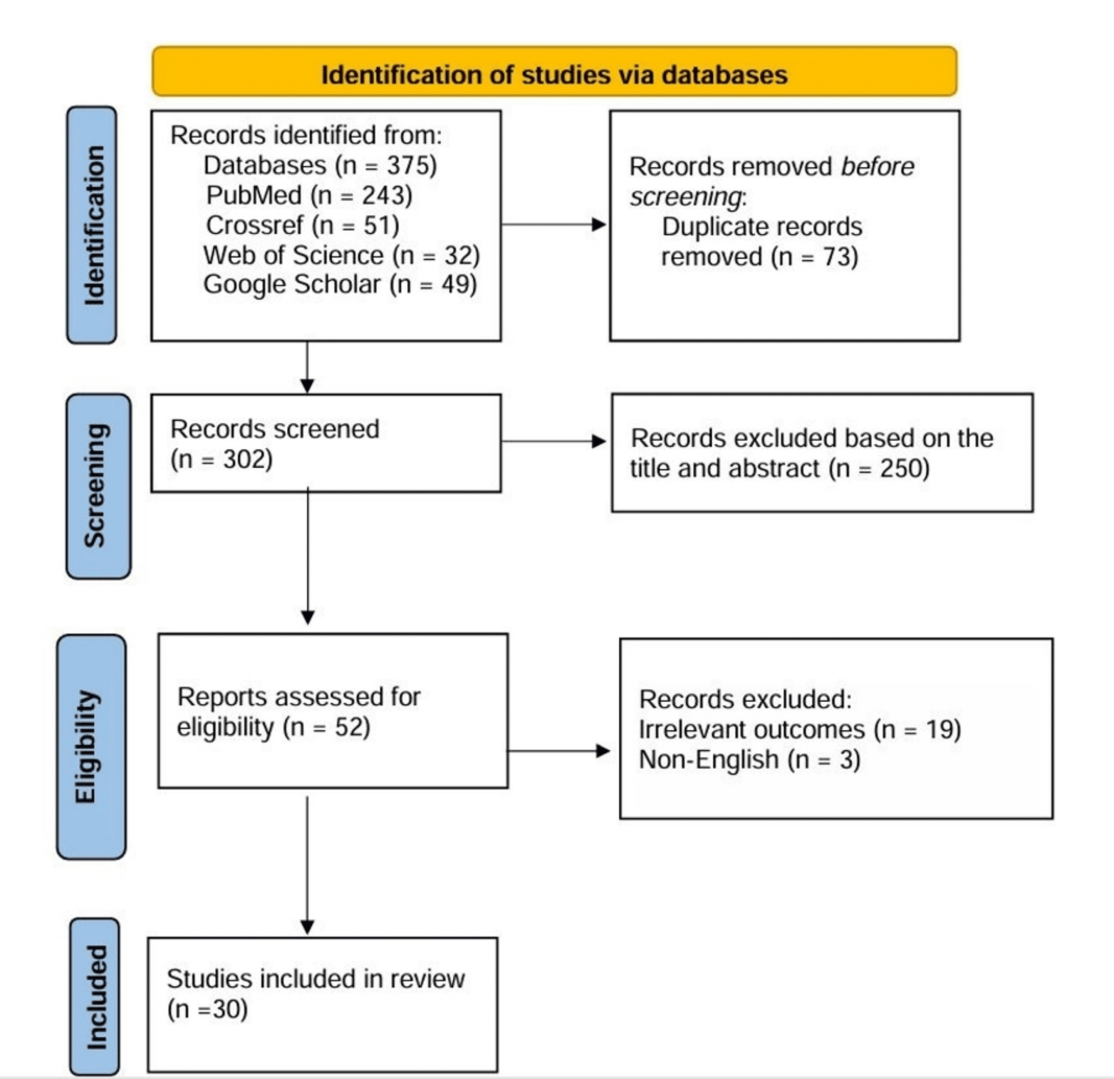
Flowchart of the study selection process

To find all eligible studies of the literature written in the English language on the relationship between germline SNPs of DNA repair genes and glioblastoma risk, we searched the database (up to January 2024) including the Cochrane Library, PubMed, Medline, and Embase, via combinations of the following terms: Brain tumors; glioma; glioblastoma; gene associations; SNPs; XRCC1 gene; XRCC3 gene; ERCC1 gene; ERCC2 gene Furthermore, reference lists of included studies were examined for additional eligible articles. The flowchart with the collection procedure for the contained studies is shown in Figure 1.

We integrated published articles between 1990 and 2023.

The following data was pulled from the study collection: authors’ information and publication year; the number of total cases and controls; the country where the study was performed; the age and gender of cases and controls; polymorphisms and DNA repair genes examined in this report; genotyping procedures; polymorphism’s genotype; the number of cases and controls; and the included odds ratios (ORs) and confidence intervals (CIs).

For XRCC1 rs25487, XRCC3 rs861539, ERCC1 rs3212986, and ERCC2 rs13181, we performed meta-analyses (Table 1).

**Table 1:** Gene, chromosome position, possible mechanisms of function, and number of studies NER: nucleotide excision repair; BER: base excision repair; HRR: homologous recombination repair; SNP: single nucleotide polymorphisms; ERCC1: excision repair cross-complementation group 1; ERCC2: excision repair cross-complementation group 2; XRCC1: X-ray repair cross-complementing protein 1; XRCC3: X-ray repair cross-complementing protein 3

Gene	SNP ID	SNP	Chromosome position	Possible mechanisms of function	Total number of studies included
ERCC1	RS3212986	C8092A	19q13.2	NER pathway	8
ERCC2	Rs13181	K751Q	19q13.3	NER	8
XRCC1	Rs25487	Arg399Gly	19q13.2	BER pathway	17
XRCC3	rs861539	T241M	14q32.3	HRR pathway	15

We enclosed data from candidate gene association studies (CGASs), and we didn’t include data from genome-wide association studies (GWASs) in any phases.

Inclusion Criteria

If a study met the following population, intervention, comparison, outcomes, and study (PICOS) diagram principles, it was suitable for insertion in the nearby meta-analysis: i) Population: limited to studies with SNPs of DNA repair genes and HGGs risk; ii) Intervention: limited to studies with gene associations and SNPs, XRCC1 gene, XRCC3 gene, ERCC1 gene, and ERCC2 gene. iii) Comparison: studies comparing the outcomes between SNPs of DNA repair genes and HGG risk; iv) The entire information of these studies is shown in Table 2 [6-14,31-50].

**Table 2:** Design and baseline characteristics of the trials included in the present meta-analysis ERCC1: excision repair cross-complementation group 1; ERCC2: excision repair cross-complementation group 2; XRCC1: X-ray repair cross-complementing protein 1; XRCC3: X-ray repair cross-complementing protein 3

Authors	Genes	Sample (cases)	Sample (controls)	mean age (cases)	mean age (controls)	Gender (cases)		Gender (controls)	
-						M	F	M	F
Wang et al., 2004 [6]	XRCC3 + XRCC1	309	342	44.1	43.8	167	142	167	175
Wrensch et al., 2005 [7]	ERCC1 + ERRC2	548	469	55	55	296	252	277	192
Felini et al., 2007 [35]	XRCC1	334	427	-	-	-	-	-	-
Kiuru et al., 2008 [8]	XRCC3 + XRCC1	320	1560	53	51.8	-	-	-	-
Liu et al., 2009 [9]	XRCC3	373	365	-	-	212	161	159	206
Zhou et al., 2009 [10]	XRCC3	771	752	-	-	462	297	463	289
McKean-Cowdin et al., 2009 [44]	ERCC2 + ERCC1 + XRCC1	1015	1994	56.3	53.6	619	396	1,020	974
Rajaraman et al., 2010 [11]	ERCC2 + XRCC3 + XRCC1	362	444	51.2	50.4	198	164	256	188
Yosunkaya et al., 2010 [13]	XRCC1	119	180	-	-	-	-	-	-
Zhou et al., 2011 [14]	XRCC1	271	289	47.8	46.9	168	103	180	109
Hu et al., 2011 [12]	XRCC1	127	249	49.5	48.9	87	40	166	83
Custódio et al., 2011 [26]	XRCC3 + XRCC1	80	100	45	45	52	28	63	37
Zhang et al., 2012 [49]	ERCC1	257	278	47.6	46.8	167	90	173	105
Liu et al., 2012 [42]	XRCC3	312	312	51.7	52.0	185	127	171	141
Chen et al., 2012 [32]	ERCC1	393	803	50.4	49.6	242	151	254	156
Luo et al., 2013 [43]	XRCC3 + XRCC1	297	415	48.7	50.2	170	127	250	165
Pan et al., 2013 [45]	ERCC1 + XRCC3 + XRCC1	443	443	50.9	51.2	257	186	257	186
Rodriguez-Hernandez et al., 2013 [46]	ERCC2 + XRCC3 + XRCC1	115	200	63.2	over 60	68	47	119	81
Salnikova et al., 2013 [47]	ERCC2	284	464	7.17	27.49	160	124	277	187
Zhao et al., 2013 [50]	XRCC3	384	384	62.4	61.5	222	162	217	167
Xu et al., 2014 [48]	XRCC3 + XRCC1	886	886	41.8	42.0±9.0	487	399	483	403
Hui et al., 2014 [40]	ERCC2 + ERCC1	138	276	45.2	44.7	85	53	170	106
Li et al., 2014 [41]	XRCC1	368	346	48.4	46.9	189	179	170	176
Gao et al., 2014 [37]	XRCC3 + XRCC1 + ERCC2 + ERCC1	326	376	47.5	48.6	194	132	224	152
Dong et al., 2014 [33]	ERCC1	72	302	44	46	44	28	119	183
Huang et al., 2015 [39]	XRCC3	389	358	48.6	47.6	199	190	174	184
Fan et al., 2016 [34]	XRCC1	115	228	48.53	46.65	66	49	124	104
Franceschi et al., 2016 [36]	XRCC3 + XRCC1	83	151	62	-	49	36	99	69
Gao et al., 2016 [38]	ERCC2	165	330	56.26	53.37	95	70	190	140
Al-Khatib et al., 2020 [31]	ERCC2	75	224	45.429	30.596	51	33	78	147

All articles that enclosed glioma cases and comprised basic ORs and CIs or the raw information required to estimate ORs and CIs were taken as suitable.

Statistical Analysis and Assessment of Heterogeneity

All investigations were extracted via Review Manager Software (RevMan), version 5.4. A random-effects model was used for the meta-analysis (in accordance with the Cochrane Handbook for Systematic Reviews of Interventions (version 5.1.0)) [12] for the assessment of the quantity calculated for each outcome separately, as the I^2^ statistic estimated the heterogeneity.

Quality Assessment

The continuous outcomes were considered as a weighted mean difference with 95% CI. A p-value of less than 0.05 was taken to indicate a statistically significant difference.

Results

Records identified through databases and after duplication removed were 287 studies. After applying all inclusion and exclusion criteria and the extraction of all full-text articles for unclear or confused results, we concluded with a quantitative synthesis with 30 articles [6,8-14,25,26,31-50].

The total number of patients was 23,678 (9,731 in cases (poor outcome) and 13,947 in controls (good outcome)). The meta-analysis pool was based on 30 studies (Table 2).

Epidemiological and Clinical Features

The mean age of the participants between the enrolled articles is presented in Table 2. The number of men and women among participants and control individuals between the eligible articles is demonstrated in Table 2.

X-ray Cross-Complementing Protein 1 (XRCC1) Gene: rs25487

**Figure 2:**
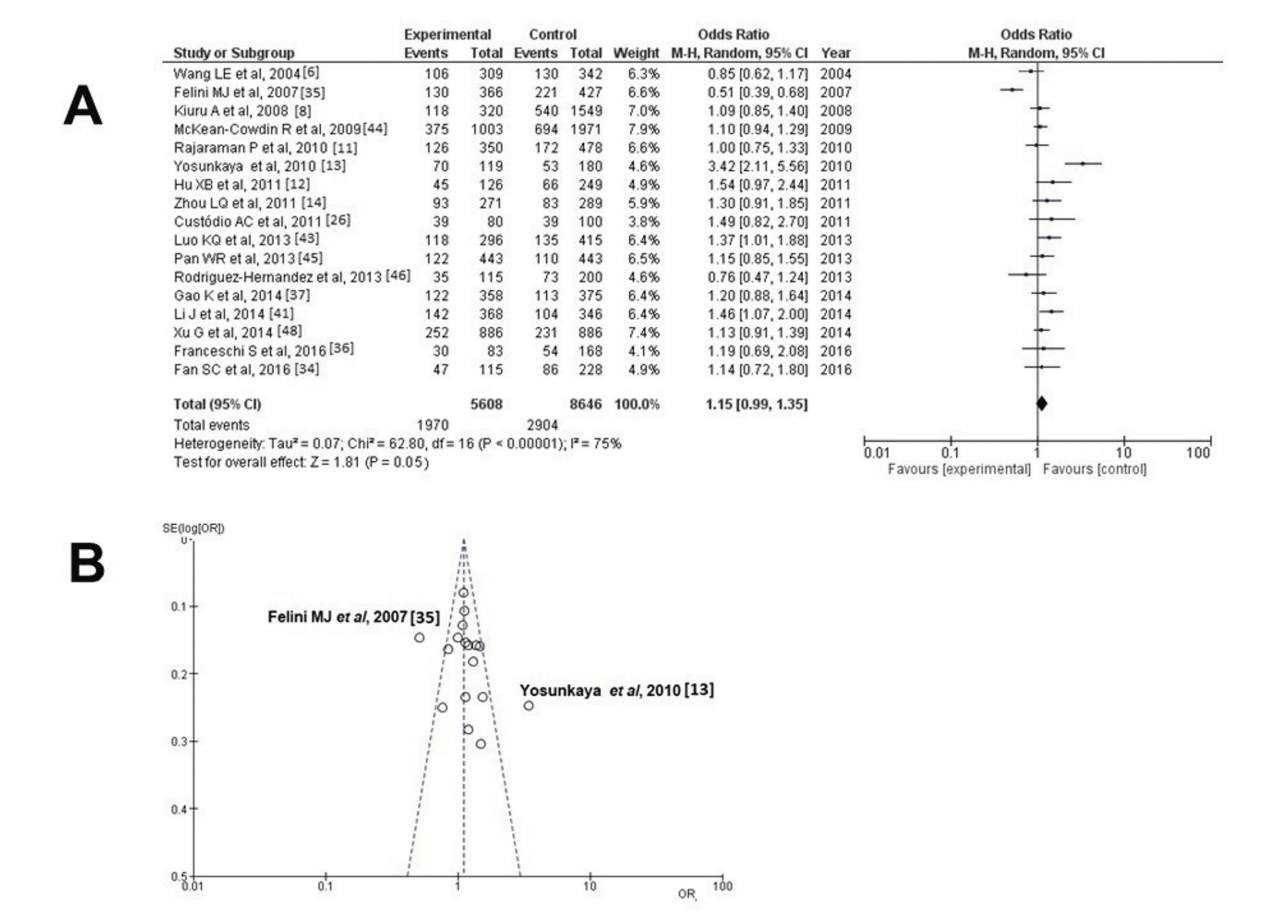
Analysis for XRCC1 - rs25487 (A) Forest plot for XRCC1 - rs25487: The results demonstrate a statistically significant difference among participants with HGGs - patients with unfavorable outcomes and controls (OR: 1.15; 95% CI: 0.99-1.35; and p = 0.05), but with heterogeneity (p < 0.05 and I^2^ = 75%); (B) Funnel plots of the XRCC1 - rs25487 with the studies by Felini et al., 2007 and Yosunkaya et al., 2010 and with heterogeneity (p < 0.05 and I^2^ = 75%)

Information regarding the SNP rs25487 parameter was available in 17 articles [6,8,11-14,26,35,37,38,41,43-46,48] and showed a statistically significant result among participants with HGGs - cases with unfavorable outcomes and controls (OR: 1.15; 95% CI: 0.99-1.35; and p = 0.05) - but with heterogeneity (p < 0.05 and I^2^ = 75%) (Figure 2A, Table 3).

**Table 3:** Results from meta-analyses of the XRCC1 rs25487, XRCC3 rs861539, ERCC1 rs3212986 and ERCC2 rs13181 for association with GBM I^2:^ percentage of total variation across studies that is due to heterogeneity rather than chance; CI: confidence interval; ERCC1: excision repair cross-complementation group 1; ERCC2: excision repair cross-complementation group 2; XRCC1: X-ray repair cross-complementing protein 1; XRCC3: X-ray repair cross-complementing protein 3

Gene	Polymorphism	Number of Studies (references)	Population	Heterogeneity		Meta-analysis model	Test for overall effect	
				I^2^	p-value		OR (95% CI)	p-value
XRCC1	rs25487	17 [6,8,11-14,26,35,37,38,41,43-46,48]	Mixed	75%	<0.05	Random	1.15 (0.99-1.35)	0.05
		14 [6,8,11,12,14,26,36,37,41,43-46,48]	Mixed	0%	0.46	Random	1.14 (1.06-1.23)	<0.05
XRCC3	rs861539	15 [6,8-11,26,36,37,39,42,43,45,46,48,50]	Mixed	37%	0.08	Random	1.09 (0.97-1.22)	0.15
ERCC1	rs3212986	8 [25,32,33,37,40,44,45,49]	Mixed	0%	0.90	Random	1.02 (0.92-1.13)	0.75
ERCC2	rs13181	8 [11,31,37,38,40,44,46,47]	Mixed	0%	0.68	Random	1.14 (1.02-1.27)	<0.05

When investigating the funnel plot, it was established that the results without the articles by Felini et al. [35] and Yosunkaya et al. [13] exhibited more preferable dispersion with a low publication bias (Figure 2B).

**Figure 3:**
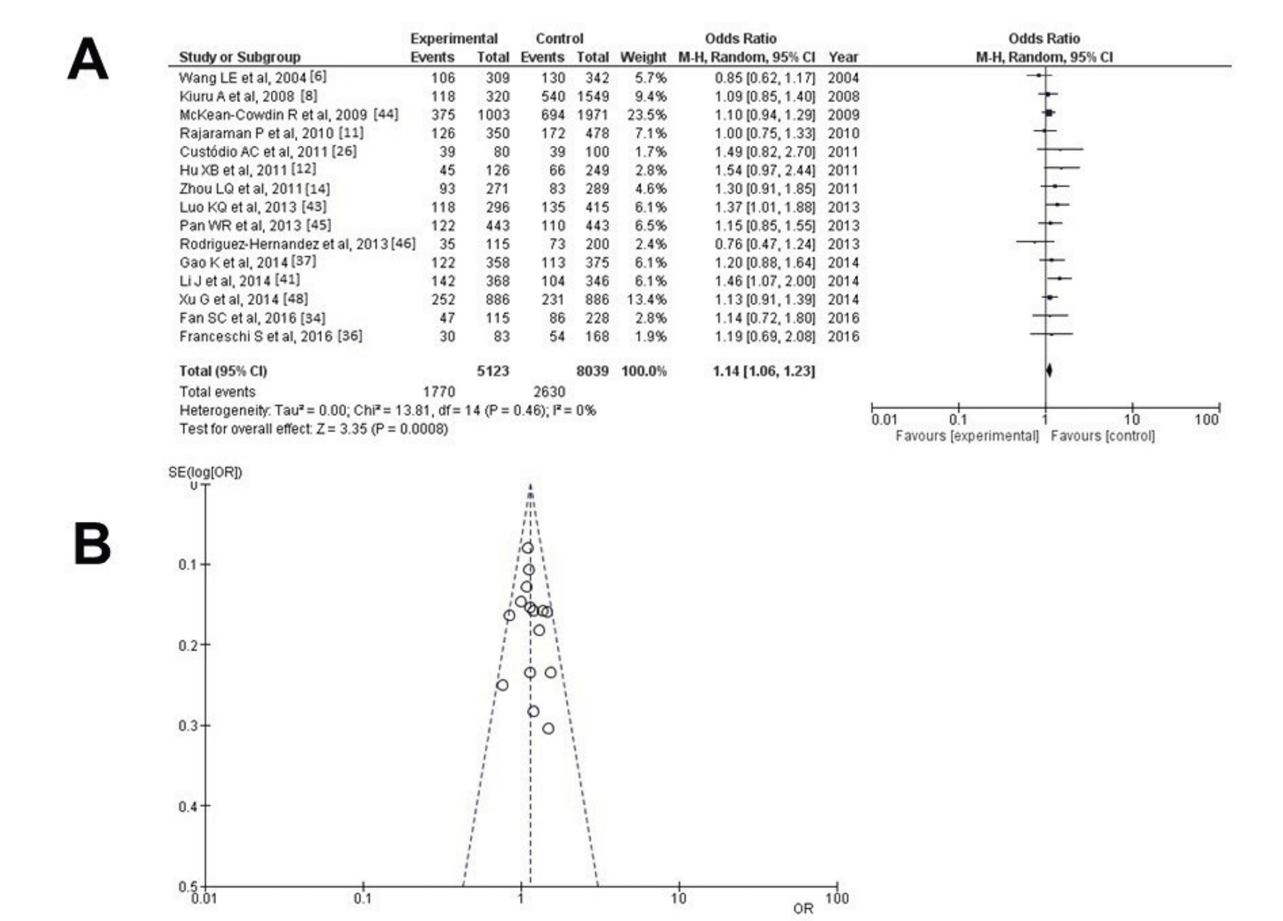
Analysis for XRCC1 - rs25487 (A) Forest plot for XRCC1 - rs25487 without the studies by Felini et al. and Yosunkaya et al. and without heterogeneity (p = 0.46 and I^2^ = 0%); (B) Forest plot for XRCC1 - rs25487 without the studies by Felini et al. and Yosunkaya et al. was found again to have a statistically significant difference (OR: 1.14; 95% CI: 1.06-1.23; p < 0.05). XRCC1: X-ray repair cross-complementing protein 1; I²: the proportion of total variation across studies that is due to heterogeneity rather than chance; CI: confidence interval; p: p-value; OR: odds ratio

No heterogeneity (p = 0.46 and I^2^ = 0%) was attained only after excluding the studies by Felini et al. [35] and Yosunkaya et al. [13]; once more, a statistically significant difference was demonstrated (OR: 1.14; 95% CI: 1.06-1.23; p < 0.05) (Figure 3A). When investigating the funnel plot, it was established that after excluding the studies by Felini et al. [35] and Yosunkaya et al. [13], it was established that the data results had a low publication bias without heterogeneity (p = 0.46 and I^2^ = 0%) (Figure 3B).

X-ray Cross-Complementing Protein 3 (XRCC3) Gene: rs861539

**Figure 4:**
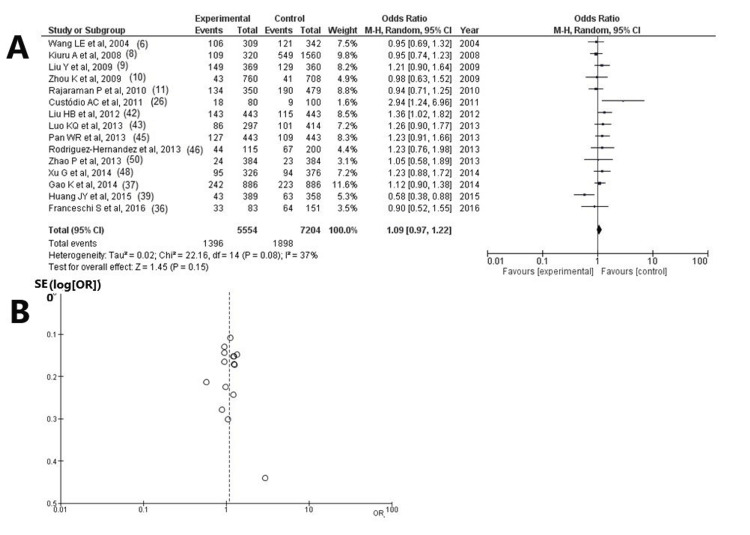
Analysis for XRCC3 - rs861539 (A) Forest plot for XRCC3 - rs861539: The results demonstrate no statistically significant difference among participants with HGGs - patients with unfavorable outcomes and controls (OR: 1.09; 95% CI: 0.97-1.22; and p = 1.15), but with low heterogeneity (p = 0.08 and I^2^ = 37%); (B) Funnel plots of the XRCC3 - rs861539 with low heterogeneity (p = 0.08 and I^2^ = 37%) and thus low publication bias XRCC3: X-ray repair cross-complementing protein 3; I²: the proportion of total variation across studies that is due to heterogeneity rather than chance; CI: confidence interval; p: p-value; OR: odds ratio

As regards SNP rs861539, information was available in 15 articles [6,8-11,26,36,37,39,42,43,45,46,48,50] and revealed no statistically significant result among the participants with HGGs-cases with unfavorable outcomes and controls (OR: 1.09; 95% CI: 0.97-1.22; and p = 1.15) (Figure 4A, Table 3). SNP rs861539 was found in 1,898 of 7,204 (26.3%) control participants and in 1,396 of 5,554 (25.1%) cases. When the funnel plot was examined, it was found that the study results revealed low heterogeneity (p = 0.08 and I2 = 37%) and a low publication bias (Figure 4B).

Excision Repair Cross-Complementation Group 1 (ERCC1) Gene: rs3212986

**Figure 5:**
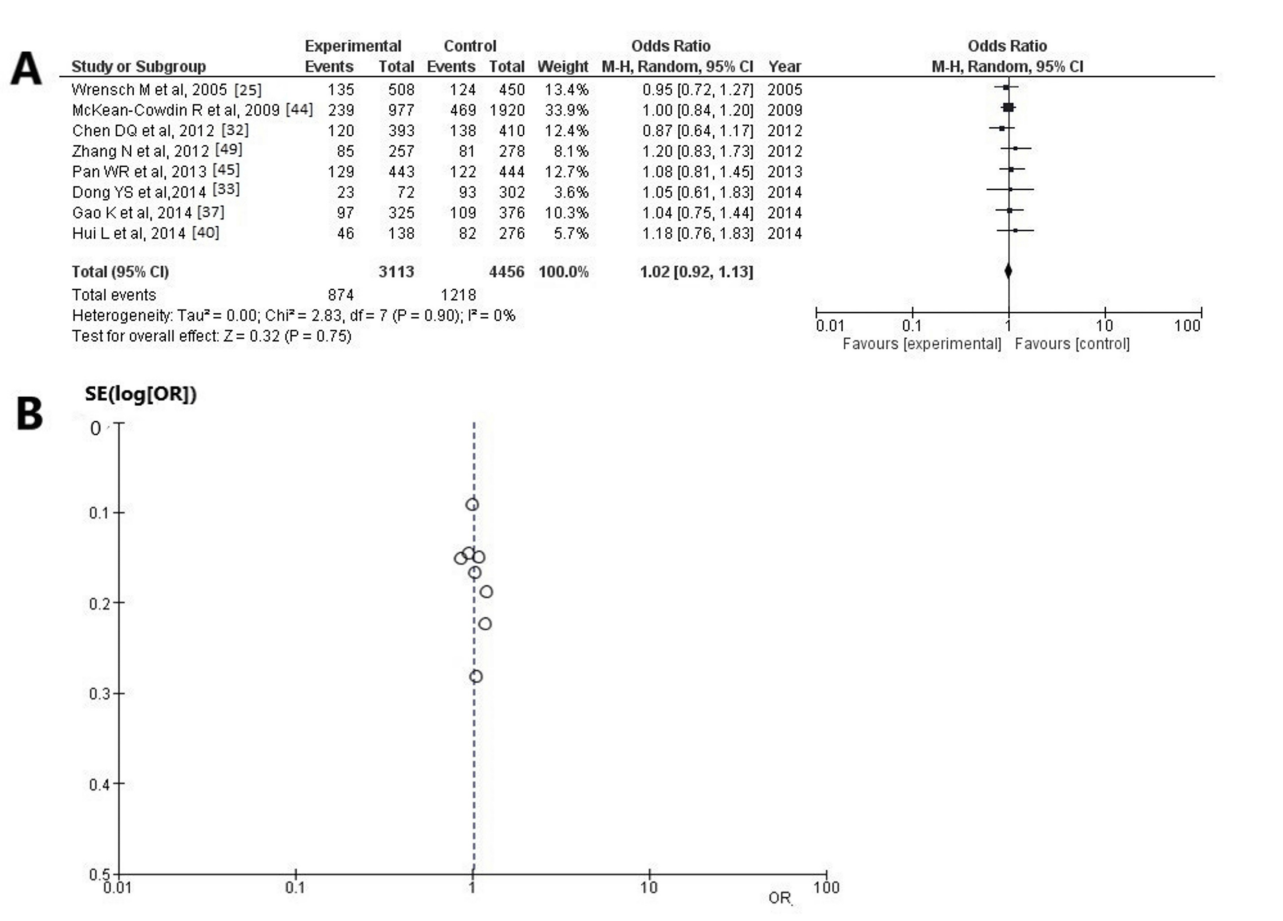
Analysis for ERCC1 - rs3212986 (A) Forest plot for ERCC1 - rs3212986: the results demonstrate no statistically significant difference between patients with HGGs - patients with unfavorable outcomes and controls (OR: 1.02; 95% CI: 0.92-1.13; and p = 0.75), and without heterogeneity (p = 0.90 and I^2^ = 0%); (B) Funnel plots of the ERCC1 - rs3212986 with no heterogeneity (p = 0.90 and I^2^ = 0%) and thus no publication bias. ERCC1: excision repair cross-complementation group 1; I²: the proportion of total variation across studies that is due to heterogeneity rather than chance; CI: confidence interval; p: p-value; OR: odds ratio

Information regarding SNP rs3212986 was available in eight articles [25,32,33,37,40,44,45,49] and exhibited no statistically significant result among the participants with HGG cases with unfavorable outcomes and controls (OR: 1.02; 95% CI: 0.92-1.13; and p = 0.75) (Figure 5A, Table 3). SNP rs3212986 was established in 1,218 of 4,456 (27.3%) control patients and 874 of 3,113 (28.0%) cases. When the funnel plot was examined, it was estimated that the study results revealed no heterogeneity (p = 0.90 and I^2^ = 0%) and no publication bias (Figure 5B).

Excision Repair Cross Complementation Group 2 (ERCC2) Gene: rs13181

**Figure 6:**
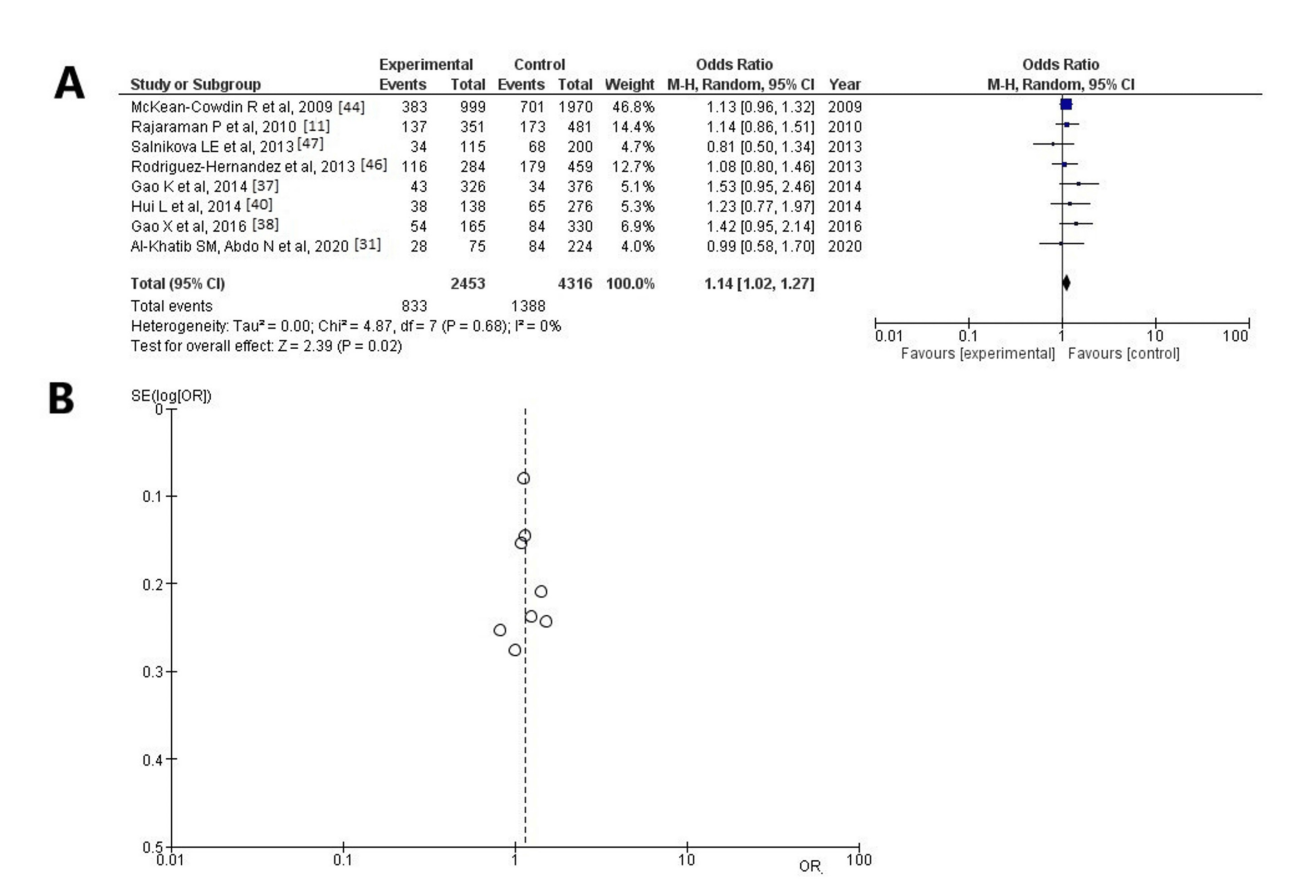
Analysis for ERCC2 - rs13181 (A) Forest plot for ERCC2 - rs13181: the results demonstrate a statistically significant difference between patients with HGGs - patients with unfavorable outcomes and controls (OR: 1.14; 95% CI: 1.02-1.27; and p < 0.05), and without heterogeneity (p = 0.68 and I^2^ = 0%); (B) Funnel plots of the ERCC2 - rs13181 with no heterogeneity (p = 0.68 and I^2^ = 0%) and thus no publication bias. ERCC2: excision repair cross-complementation group 2; I²: the proportion of total variation across studies that is due to heterogeneity rather than chance; CI: confidence interval; p: p-value; OR: odds ratio

As regards SNP rs13181, information was available in eight articles [11,31,37,38,40,44,46,47] and showed a statistically significant result among the participants with HGG cases with unfavorable outcomes and controls (OR: 1.14; 95% CI: 1.02-1.27; and p < 0.05) (Figure 6A, Table 3). SNP rs13181 was found in 1,388 of 4,316 control participants and in 833 of 2,453 cases. When the funnel plot was examined, it was found that the study results revealed no heterogeneity (p = 0.68 and I^2^ = 0%) and no publication bias (Figure 6B).

Discussion

Glioma prevalence as a primary brain tumor is almost 80%, and it is still a relatively rare entity [51], which made it difficult for any single study to recruit the appropriate number of patients to strengthen the genetic association analyses. This may partly explain the inconclusive results from studies searching for this genetic association on different genes, polymorphisms, and glioma risk. We collected eligible studies together to evaluate the association between ERCC1, ERCC2, XRCC1, and XRCC3 polymorphisms and glioma risk in the present study. We searched for certain SNPs: ERCC1 (rs3212986), ERCC2 (rs13181), XRCC1 (rs25487), and XRCC3 (rs861539). In the current meta-analysis, we established a significant relationship between rs25487 and rs13181 and glioma susceptibility.

Over the past years, many authors have shown that certain genetic variations in key genes involved in DNA repair pathways can lead to an increased risk of developing cancer, while others have suggested that these same variations may actually confer some protective effects against certain types of cancer. Numerous exogenous or endogenous factors can cause DNA damage, necessitating effective DNA repair to restore genomic integrity and involving numerous DNA genes [52,53]. Changes in ERCC1, ERCC2, XRCC1, and XRCC3 may potentially influence cancer development by disturbing the DNA repair mechanism [40,52,53]. Also, DNA repair genes (like XRCC1) that protein creation is occupied in a number of repair pathways are polymorphic and important to distorted capability for base excision-repair (BER) in answer to genotoxic agents such as ionizing radiation and chemical mediators [52]. These genotoxic mediators could change organic particles together with DNA by direct or indirect ways of the production of reactive oxygen species (ROS) [54]. In literature, cells, mainly in eukaryotic organisms, react to radiation by numerous DNA injury repair pathways and by removing harmful chemicals from the ROS through certain antioxidant methods [55]. For example, base excision repair needs activation of the XRCC1. Furthermore, XRCC3 is necessitated for homologous recombination DNA repair (HRR) [56].

Wang et al. [6] were the first to explore the association between XRCC1 polymorphism and glioma susceptibility, and many authors have been trying to investigate the connection since then [6,8,12,14,26,35]. ERCC1 and ERCC2 proteins are crucial for capable DNA repair ability, as they are significant parts of the transcription-coupled nucleotide excision repair (NER) pathway. They are essential for the preservation of genetic constancy [53]. During NER, the ERCC1 gene codes for a protein that creates the 5’ cut by forming a complex with XPF. In addition, mutations in the ERCC2 gene were found to affect the DNA repair proficiency [53].

Deficiency in ERCC1 and ERCC2 proteins is connected with the risk of malignancies [57]. Research has shown that individuals with mutations or lower expression levels of ERCC1 and ERCC2 are at an increased risk of developing certain types of cancers, emphasizing the important role these proteins play in maintaining genomic stability and preventing the formation of tumors [57]. Further studies are needed to fully understand the connection between ERCC1 and ERCC2 deficiencies and cancer risk, as well as to explore potential therapeutic strategies for individuals with these deficiencies.

Deficiency in ERCC1 and ERCC2 can lead to a higher accumulation of DNA damage, which increases the likelihood of mutations that can ultimately lead to cancer development. Additionally, individuals with deficiencies in these proteins may be more susceptible to the harmful effects of genotoxic agents such as chemotherapy drugs.

Our meta-analysis included 30 articles and thus constituted a wide-ranging evaluation of the relationships of ERCC1 C8092A, ERCC2 Lys751Gln, XRCC1 Arg399Gly, and XRCC3 T241M polymorphisms with the risk of glioma in various populations. It included more than 23,678 glioma patients: 3,113 cases and 4,456 controls for rs3212986; 2,453 cases and 4,316 controls for rs13181; 5,123 cases and 8,039 controls for rs25487; and for rs861539, it included 5,554 cases and 7,204 controls.

This study has several limitations. As most of the evaluated SNPs have been examined in small studies, most did not show statistically significant correlations due to their little statistical power. Additionally, our purpose was to present any proof of the connection between the SNPs and glioma susceptibility rather than to explore the paths following these involvements. Also, the lack of potential moderators, such as tumor origin and the differentiation to high-grade or low-grade gliomas, could make this study more valuable. Finally, the results of XRCC3 had low publication bias. Thus, a screening test that will include the ERCC1 (rs3212986), ERCC2 (rs13181), XRCC1 (rs25487), and XRCC3 (rs861539) genes may well be helpful for the prevention and earliest treatment of patients with glioma susceptibility. More original gene-associated studies are needed to detect the mechanisms through which these polymorphisms manipulate cancer vulnerability.

This comprehensive meta-analysis declared a significant association between ERCC2 rs13181, XRCC1 rs25487, and the risk of glioma. Further studies with a larger sample size and more ethnic groups are advised to elucidate the possible roles of other genes in the etiology and progression of glioma. Additionally, clinical protocol implies screening proposed genes associated with glioma etiology and outcome, which should be amplified in more institutions for a widespread study of the genes associated with glioma for early discovery and better outcomes.

## Conclusions

This comprehensive meta-analysis declared a significant association between ERCC2 rs13181, XRCC1 rs25487, and the risk of glioma. Further studies with a larger sample size and more ethnic groups are advised to elucidate the possible roles of other genes in the etiology and progression of glioma. Additionally, clinical protocol implies screening proposed genes associated with glioma etiology and outcome, which should be amplified in more institutions for a widespread study of the genes associated with glioma for early discovery and better outcomes.

## References

[1] Ohgaki H, Kleihues P (2005). Epidemiology and etiology of gliomas. Acta Neuropathol.

[2] Mofatteh M, Arfaie S, Mashayekhi MS, Pearl PL, Das S, Cohen-Gadol A (2024). Editorial: seizures in brain tumors. Front Surg.

[3] Louis DN, Perry A, Reifenberger G (2016). The 2016 World Health Organization classification of tumors of the central nervous system: a summary. Acta Neuropathol.

[4] Louis DN, Perry A, Wesseling P (2021). The 2021 WHO classification of tumors of the central nervous system: a summary. Neuro Oncol.

[5] de Tayrac M, Saikali S, Aubry M, Bellaud P, Boniface R, Quillien V, Mosser J (2013). Prognostic significance of EDN/RB, HJURP, p60/CAF-1 and PDLI4, four new markers in high-grade gliomas. PLoS One.

[6] Wang LE, Bondy ML, Shen H (2004). Polymorphisms of DNA repair genes and risk of glioma. Cancer Res.

[7] Liu W, Long H, Zhang M (2019). Glutathione S-transferase genes variants and glioma risk: a case-control and meta-analysis study. J Cancer.

[8] Kiuru A, Lindholm C, Heinävaara S (2008). XRCC1 and XRCC3 variants and risk of glioma and meningioma. J Neurooncol.

[9] Liu Y, Scheurer ME, El-Zein R (2009). Association and interactions between DNA repair gene polymorphisms and adult glioma. Cancer Epidemiol Biomarkers Prev.

[10] Sarwar R, Mahjabeen I, Bashir K, Saeed S, Kayani MA (2017). Haplotype based analysis of XRCC3 gene polymorphisms in thyroid cancer. Cell Physiol Biochem.

[11] Rajaraman P, Hutchinson A, Wichner S (2010). DNA repair gene polymorphisms and risk of adult meningioma, glioma, and acoustic neuroma. Neuro Oncol.

[12] Hu XB, Feng Z, Fan YC, Xiong ZY, Huang QW (2011). Polymorphisms in DNA repair gene XRCC1 and increased genetic susceptibility to glioma. Asian Pac J Cancer Prev.

[13] Yosunkaya E, Kucukyuruk B, Onaran I, Gurel CB, Uzan M, Kanigur-Sultuybek G (2010). Glioma risk associates with polymorphisms of DNA repair genes, XRCC1 and PARP1. Br J Neurosurg.

[14] Zhou LQ, Ma Z, Shi XF, Yin XL, Huang KX, Jiu ZS, Kong WL (2011). Polymorphisms of DNA repair gene XRCC1 and risk of glioma: a case-control study in Southern China. Asian Pac J Cancer Prev.

[15] Yuan G, Gao D, Ding S, Tan J (2014). DNA repair gene ERCC1 polymorphisms may contribute to the risk of glioma. Tumour Biol.

[16] Wang L, Jiang YQ, Zhou MD, Jiang Z (2015). Association between XRCC1 Arg399Gln polymorphism and glioma risk in a Chinese population: a case-control study. Int J Clin Exp Med.

[17] Liu H, Mao P, Xie C, Xie W, Wang M, Jiang H (2015). Association between interleukin 8-251 T/A and +781 C/T polymorphisms and glioma risk. Diagn Pathol.

[18] Zhou Q, Fu Y, Wen L, Deng Y, Chen J, Liu K (2021). XPD polymorphisms and risk of hepatocellular carcinoma and gastric cancer: a meta-analysis. Technol Cancer Res Treat.

[19] Zhu H, Cao S, Liu Y, Ding X, Wu Q, Ma H (2013). Genetic polymorphisms of xeroderma pigmentosum group D and prostate cancer risk: a meta-analysis. J Cancer Res Ther.

[20] Li W, Li K, Zhao L, Zou H (2014). DNA repair pathway genes and lung cancer susceptibility: a meta-analysis. Gene.

[21] Ramaniuk VP, Nikitchenko NV, Savina NV (2014). Polymorphism of DNA repair genes OGG1, XRCC1, XPD and ERCC6 in bladder cancer in Belarus. Biomarkers.

[22] Yang QI, Wei YF, Zhang Y, Huang GM (2015). XPD Lys(751)Gln and Asp(312)Asn polymorphisms and hepatocellular carcinoma susceptibility: a meta-analysis of 11 case-control studies in an Asian population. Exp Ther Med.

[23] Rengifo Rojas C, Cercy J, Perillous S (2024). Biallelic non-productive enhancer-promoter interactions precede imprinted expression of Kcnk9 during mouse neural commitment. HGG Adv.

[24] Nothnagel M, Ellinghaus D, Schreiber S, Krawczak M, Franke A (2009). A comprehensive evaluation of SNP genotype imputation. Hum Genet.

[25] Wrensch M, Kelsey KT, Liu M (2005). ERCC1 and ERCC2 polymorphisms and adult glioma. Neuro Oncol.

[26] Custódio AC, Almeida LO, Pinto GR (2011). Analysis of the polymorphisms XRCC1Arg194Trp and XRCC1Arg399Gln in gliomas. Genet Mol Res.

[27] Yao L, Ji G, Gu A, Zhao P, Liu N (2012). An updated pooled analysis of glutathione S-transferase genotype polymorphisms and risk of adult gliomas. Asian Pac J Cancer Prev.

[28] Cao W, Xiong L, Meng L (2023). Prognostic analysis and nomogram construction for older patients with IDH-wild-type glioblastoma. Heliyon.

[29] Ohgaki H, Dessen P, Jourde B (2004). Genetic pathways to glioblastoma: a population-based study. Cancer Res.

[30] Houillier C, Lejeune J, Benouaich-Amiel A (2006). Prognostic impact of molecular markers in a series of 220 primary glioblastomas. Cancer.

[31] Al-Khatib SM, Abdo N, Al-Eitan LN, Al-Mistarehi AW, Zahran DJ, Al Ajlouni M, Kewan TZ (2020). The Impact of the genetic polymorphism in DNA repair pathways on increased risk of glioblastoma multiforme in the Arab Jordanian population: a case-control study. Appl Clin Genet.

[32] Chen DQ, Yao DX, Zhao HY, Yang SJ (2012). DNA repair gene ERCC1 and XPD polymorphisms predict glioma susceptibility and prognosis. Asian Pac J Cancer Prev.

[33] Dong YS, Hou WG, Li XL (2014). Genetic association of CHEK2, GSTP1, and ERCC1 with glioblastoma in the Han Chinese population. Tumour Biol.

[34] Fan SC, Zhou JG, Yin JZ (2016). Investigation of the role of XRCC1 genetic polymorphisms in the development of gliomas in a Chinese population. Genet Mol Res.

[35] Felini MJ, Olshan AF, Schroeder JC (2007). DNA repair polymorphisms XRCC1 and MGMT and risk of adult gliomas. Neuroepidemiology.

[36] Franceschi S, Tomei S, Mazzanti CM (2016). Association between RAD 51 rs1801320 and susceptibility to glioblastoma. J Neurooncol.

[37] Gao K, Mu SQ, Wu ZX (2014). Investigation of the effects of single-nucleotide polymorphisms in DNA repair genes on the risk of glioma. Genet Mol Res.

[38] Gao X, Tang YJ, Zhang GF, Yu L, Qi ST (2016). ERCC2 rs13181 polymorphism association with glioma susceptibility in a Chinese population. Genet Mol Res.

[39] Huang JY, Yang JF, Qu Q (2015). DNA repair gene XRCC3 variants are associated with susceptibility to glioma in a Chinese population. Genet Mol Res.

[40] Hui L, Yue S, Gao G, Chang H, Li X (2014). Association of single-nucleotide polymorphisms in ERCC1 and ERCC2 with glioma risk. Tumour Biol.

[41] Li J, Qu Q, Qu J (2014). Association between XRCC1 polymorphisms and glioma risk among Chinese population. Med Oncol.

[42] Liu HB, Peng YP, Dou CW, Su XL, Gao NK, Tian FM, Bai J (2012). Comprehensive study on associations between nine SNPs and glioma risk. Asian Pac J Cancer Prev.

[43] Luo KQ, Mu SQ, Wu ZX, Shi YN, Peng JC (2013). Polymorphisms in DNA repair genes and risk of glioma and meningioma. Asian Pac J Cancer Prev.

[44] McKean-Cowdin R, Barnholtz-Sloan J, Inskip PD (2009). Associations between polymorphisms in DNA repair genes and glioblastoma. Cancer Epidemiol Biomarkers Prev.

[45] Pan WR, Li G, Guan JH (2013). Polymorphisms in DNA repair genes and susceptibility to glioma in a chinese population. Int J Mol Sci.

[46] Rodriguez-Hernandez I, Perdomo S, Santos-Briz A, Garcia JL, Gomez-Moreta JA, Cruz JJ, Gonzalez-Sarmiento R (2014). Analysis of DNA repair gene polymorphisms in glioblastoma. Gene.

[47] Salnikova LE, Belopolskaya OB, Zelinskaya NI, Rubanovich AV (2013). The potential effect of gender in CYP1A1 and GSTM1 genotype-specific associations with pediatric brain tumor. Tumour Biol.

[48] Xu G, Wang M, Xie W, Bai X (2014). DNA repair gene XRCC3 Thr241Met polymorphism and susceptibility to glioma: a case-control study. Oncol Lett.

[49] Zhang N, Lin LY, Zhu LL (2012). ERCC1 polymorphisms and risk of adult glioma in a Chinese population: a hospital-based case-control study. Cancer Invest.

[50] Zhao P, Zou P, Zhao L, Yan W, Kang C, Jiang T, You Y (2013). Genetic polymorphisms of DNA double-strand break repair pathway genes and glioma susceptibility. BMC Cancer.

[51] Schwartzbaum JA, Fisher JL, Aldape KD, Wrensch M (2006). Epidemiology and molecular pathology of glioma. Nat Clin Pract Neurol.

[52] Ginsberg G, Angle K, Guyton K, Sonawane B (2011). Polymorphism in the DNA repair enzyme XRCC1: utility of current database and implications for human health risk assessment. Mutat Res.

[53] Qian T, Zhang B, Qian C, He Y, Li Y (2017). Association between common polymorphisms in ERCC gene and glioma risk: a meta-analysis of 15 studies. Medicine (Baltimore).

[54] Buergers R, Rosentritt M, Handel G (2007). Bacterial adhesion of Streptococcus mutans to provisional fixed prosthodontic material. J Prosthet Dent.

[55] Hoeijmakers JH (2001). Genome maintenance mechanisms for preventing cancer. Nature.

[56] Bassi C, Xavier Dj, Palomino G, Nicolucci P, Soares C, Sakamoto-Hojo E, Donadi E (2008). Efficiency of the DNA repair and polymorphisms of the XRCC1, XRCC3 and XRCC4 DNA repair genes in systemic lupus erythematosus. Lupus.

[57] Goode EL, Ulrich CM, Potter JD (2002). Polymorphisms in DNA repair genes and associations with cancer risk. Cancer Epidemiol Biomarkers Prev.

